# Advances in Cancer Metabolism and Tumour Microenvironment

**DOI:** 10.3390/ijms23084071

**Published:** 2022-04-07

**Authors:** Karel Smetana, Michal Masařík

**Affiliations:** 1 Institute of Anatomy, First Faculty of Medicine, Charles University, 128 00 Prague, Czech Republic; 2 Biotechnology and Biomedicine Center, Academy of Science and Charles University, 252 50 Vestec, Czech Republic; 3 Department of Pathological Physiology, Faculty of Medicine, Masaryk University, Kamenice 5, 625 00 Brno, Czech Republic

Cancer represents an extremely complicated ecosystem where cancer cells communicate with non-cancer cells present in the tumour niche through intercellular contacts, paracrine production of bioactive factors and extracellular vesicles, such as exosomes. The non-cancer cells that participate in the control of biological properties of the malignant disease include cancer-associated fibroblasts (CAFs), tumour-associated macrophages, granulocytes, NK cells and subsets of lymphocytes [1]. Differences in the metabolism of cancer cells were established many years ago, and the relationship between the tumour microenvironment (TME) and the metabolism was shown [2,3,4]. Distinguished scientists have prepared highly instructive articles on this topic with the aim of gaining a deeper insight into the role of the microenvironment and the differences in the cancer cell metabolism to refine the diagnostics and propose new strategies for anticancer therapy. Tumour cells share the common ability to obtain necessary nutrients from a relatively poor environment and use them flexibly to maintain their viability and build new biomass. Bioenergetically demanding processes accompanying malignant transformation, such as rapid proliferation and the ability to migrate, require increased production of adenosine triphosphate (ATP), nucleic acids, proteins and lipids. The changes in intracellular and extracellular metabolites that accompany the metabolic reprogramming associated with tumour growth subsequently have a profound effect on gene expression, cellular differentiation and the tumour microenvironment [5].

Over the course of carcinogenesis, tumour cells face selection pressures that force them to continuously optimize dominant metabolic pathways, and tumour cells thus undergo major metabolic reorganizations. In general, greater flexibility of the metabolic pathways utilized by a given cell or group of cells increases their ability to match metabolic needs to a changing environment. Metabolic plasticity is mediated by a number of mechanisms involving receptors, different types of signalling pathways, altered transcription factor activity and mutations affecting enzyme expression or function. Metabolic plasticity is not an exclusive feature of cancer cells, as it plays an important role in embryogenesis. Early embryos are initially dependent on oxidative metabolism and preferentially oxidize pyruvate using maternal mitochondria from the oocyte. Glucose uptake gradually increases in the morula and is accelerated at the blastocyst stage, where its uptake exceeds that of pyruvate or lactate. Glycolysis predominates in this period and is further accelerated when the embryo is implanted into the uterine wall. During later development, mitochondrial replication allows the re-initiation of the oxidative metabolism and a progressive decline in glycolysis. These metabolic shifts are critical; mutations in genes associated with glycolysis or oxidative phosphorylation (OXPHOS) can lead to developmental delay or embryonic lethality [6]. The metabolic plasticity of embryonic cells can be “unlocked” during carcinogenesis and exploited by tumour cells.

Glioblastoma is a tumour with an extremely poor prognosis concerning patient recovery and very low survival. Cells of advanced glioblastoma are under the influence of the tumour microenvironment that is (in addition to usual elements such as CAF-like cells or immune cells) also formed by non-transformed glial cells (microglia, astrocytes). They are able to influence the metabolism of cancer cells, for example, by transferring mitochondria to tumour elements. Glial cells are able to participate in the control of glioblastoma cell metabolism (oxidative phosphorylation and production of ATP) [7]. Glioblastoma cells, namely those of the so-called mesenchymal type, highly express fibroblast activation protein (FAP). The expression of FAP was observed in cancer cells as well as in stromal elements forming the cancer microenvironment (such as mesenchymal cells, pericytes and endothelial cells). FAP expression in glioblastoma stem cells is usually lower than in other types of glioblastoma cells. The expression of FAP is dependent on TGF-β1 production by glioblastoma cells. TGF-β1 is recognized by the TGF-β type I receptor via Smad2 signalling [8].

FAP is an important molecule on the surface of CAFs that are known as modulators of the biological properties of head and neck cancer [9]. In the presented study, CAFs prepared from head and neck cancer promoted forming of colonies of cell lines prepared from this type of cancer. CAFs significantly stimulated the expression of *PGE2S*, *EGFR*, *CAV1*, *NFKB*, *FOLR1*, *COX2*, *BCL2*, *VEGFA* and *POU5F* genes that are associated with tumour progression. The tumour type-specific CAFs also significantly stimulated the resistance of cancer cells to cisplatin. Transcription analysis demonstrated that the resistance against cisplatin depends on the activity of genes *VEGFA*, *PGE2S*, *COX2*, *EGFR* and *NANOG.* The sensitivity to cisplatin can be returned by activation of the *CCL2* gene [10]. 

Mutations in the *BRCA1* gene are known to increase the risk of breast/ovarian cancer in the affected women. Extensive data about the role of these mutations in cancer cells are available, but only limited knowledge exists on the role of these mutations in the function of the tumour microenvironment. Portier and co-workers [11] prepared induced mesenchymal stem cells from patients carrying a germline deletion of exon 17 of the *BRCA1* gene. Compared to non-deficient cells, they exhibited proangiogenic gene signature (active *HIF-1α*, *VEGF*, *PDGF*, *ANGPT*) that was verified by the tube-like formation in vitro. The application of these cells with 4T1 cancer cells to the mammary fat pad of the mouse xenogeneic model demonstrated a positive effect of *BRCA1*-deficient stroma on cancer growth and the formation of vessels in the tumour site. Ovarian cancer is dependent on oestrogen-related pathways. Complex analysis of clinical material clearly demonstrated the dysregulation of the expression of genes for oestrogen receptors 1 and 2 (*ESR1/2*) and their co-regulators, such as proline-, glutamic acid- and leucine-rich protein 1 (PELP1), and proto-oncogene tyrosine-protein kinase c-Src during the ovarian cancer initiation and progression. The results demonstrated that PEPL1 is strongly upregulated at the protein level and can be employed as a diagnostic marker or for cancer targeting [12]. Steroids are also at the centre of interest in the report by Valko-Rokytská and co-workers [13], who summarize data about the synthesis, function and detection of steroids in distinct biological fluids (urine, serum, tissue, saliva, blood). They compared different analytical procedures focused predominantly on breast cancer.

Differences in the cancer cell metabolism are the result of genetic alterations responsible for malignant transformation. The mutual relations between genes and metabolic differences are summarized in the paper by Di Greogorio and colleagues [14]. They demonstrated and explained the metabolism’s Warburg alteration in light of modern powerful molecular genetic tools and used cancer as an example of metabolic disease. Radiation and immunotherapy represent powerful tools for cancer treatment. Experimental irradiation is used for the induction of senescence in cancer cells. An in vitro study employing metabolomic approaches clearly demonstrated that radiation-induced senescent colon cancer cells enhanced the expression of both pro-inflammatory IL-1 and anti-inflammatory factors, such as IL-27. The reduction in angiogenic factor VEGF-A production was also noted. From the metabolic point of view, the activity of senescent cells depends on aerobic glycolysis. These data are fundamental for better understanding the metabolism of senescent cells in tumours and after radiotherapy [15]. Cells of the immune system infiltrate solid tumours, but, unfortunately, instead of the anticancer response, they support tumour spreading and immune escape of cancer cells. Yan and colleagues [16] summarize data on how to influence the metabolism of cancer cells and immune cells to improve the efficiency of cancer immunotherapy, which represents one of the most prominent approaches to anticancer therapy. The manipulation of the cancer cell metabolism is also reviewed by Nenkov and collaborators [17], who discuss the possibility of manipulating the abnormal metabolism of cancer cells with respect to hypoxia in the cancer niche. They also present compounds suitable for targeting glucose, glutamine and lipid metabolism and their molecular effect, which could be employed for anticancer therapy. A good example of the therapeutic effect of cancer cell metabolic reprogramming is the ductal adenocarcinoma of the pancreas, where the combination of metabolic reprogramming can improve the efficiency of targeting KRAS in these patients [18].

The ectopic/aberrant expression of proteins in cancer cells can represent a very important problem for oncological patients. The high expression and activity of nicotinamide N-methyltransferase is commonly observed in various types of cancer. This enzyme is responsible for the methylation of nicotinamide and controls many metabolic pathways in cancer cells. The relation of this enzyme to cancer cell plasticity (similar to stem cell) and resistance to therapy is discussed with the goal of developing new anticancer therapy procedures [19]. Another example is the amyloid precursor protein (APP), which is known to be involved in the pathogenesis of Alzheimer’s disease. APP is also expressed in cells other than neurons and especially in cancer cells. The dysregulation of activity encoding this protein and aberrant expression of APP seem to influence important properties of cancer cells, such as metastasizing, proliferation, local invasion and drug resistance [20].

Studies collected in this Special Issue have clearly demonstrated the effect of the tumour microenvironment on the biology of cancer cells and its influence on cancer cell metabolism. This part of cancer biology is highly important because some of the intermediates of tumour metabolism can significantly influence the resulting phenotype of the tumour cells themselves and other cells present in the tumour microenvironment (TME). The most common features of tumour metabolism include: (1) deregulated uptake of glucose, acetate, citrate and some amino acids, (2) use of glycolysis, fermentation or Krebs cycle intermediates for biosynthesis, NADPH production and NAD+ regeneration, (3) increased nitrogen demand, (4) epigenetic changes caused by the accumulation of oncogenic metabolites, (5) emergence of metabolic symbiosis and use of the metabolites to manipulate TME, and (6) use of opportunistic nutrient acquisition pathways. While some tumours exhibit all of the above features, many tumour types express only a few. The specific metabolic features exhibited by each tumour type may help to better classify the tumour and aid in the selection of appropriate treatment.

A deep understanding of the cancer microenvironment and metabolic changes in cancer cells can improve our knowledge, potentially resulting in new therapeutic modalities.
